# Determination of Plasmid pSN1216-29 Host Range and the Similarity in Oligonucleotide Composition Between Plasmid and Host Chromosomes

**DOI:** 10.3389/fmicb.2020.01187

**Published:** 2020-06-09

**Authors:** Maho Tokuda, Haruo Suzuki, Kosuke Yanagiya, Masahiro Yuki, Kengo Inoue, Moriya Ohkuma, Kazuhide Kimbara, Masaki Shintani

**Affiliations:** ^1^Applied Chemistry and Biochemical Engineering Course, Department of Engineering, Graduate School of Integrated Science and Technology, Shizuoka University, Shizuoka, Japan; ^2^Institute for Advanced Biosciences, Keio University, Tsuruoka, Japan; ^3^Faculty of Environment and Information Studies, Keio University, Fujisawa, Japan; ^4^Japan Collection of Microorganisms, RIKEN BioResource Research Center, Tsukuba, Japan; ^5^Faculty of Agriculture, University of Miyazaki, Miyazaki, Japan; ^6^Research Institute of Green Science and Technology, Shizuoka University, Shizuoka, Japan

**Keywords:** plasmid, conjugation, host range, oligonucleotide composition, pSN1216-29

## Abstract

Plasmids are extrachromosomal DNA that can be horizontally transferred between different bacterial cells by conjugation. Horizontal gene transfer of plasmids can promote rapid evolution and adaptation of bacteria by imparting various traits involved in antibiotic resistance, virulence, and metabolism to their hosts. The host range of plasmids is an important feature for understanding how they spread in environmental microbial communities. Earlier bioinformatics studies have demonstrated that plasmids are likely to have similar oligonucleotide (*k*-mer) compositions to their host chromosomes and that evolutionary host ranges of plasmids could be predicted from this similarity. However, there are no complementary studies to assess the consistency between the predicted evolutionary host range and experimentally determined replication/transfer host range of a plasmid. In the present study, the replication/transfer host range of a model plasmid, pSN1216-29, exogenously isolated from cow manure as a newly discovered self-transmissible plasmid, was experimentally determined within microbial communities extracted from soil and cow manure. *In silico* prediction of evolutionary host range was performed with the pSN1216-29 using its oligonucleotide compositions independently. The results showed that oligonucleotide compositions of the plasmid pSN1216-29 had more similarities to those of hosts (transconjugants genera) than those of non-hosts (other genera). These findings can contribute to the understanding of how plasmids behave in microbial communities, and aid in the designing of appropriate plasmid vectors for different bacteria.

## Introduction

Plasmids are extrachromosomal elements in bacterial cells, which can carry accessory genes including catabolic and antibiotic resistance genes. Horizontal gene transfer (HGT) by conjugative plasmid promotes rapid bacterial evolution and adaptation in natural environments (Ramirez et al., 2014). Host ranges of plasmids are one of the essential features for understanding how the plasmids could spread in natural environments. Generally, a host range is estimated qualitatively (as ‘narrow’ or ‘broad’) and determined using conjugation assays between one donor strain and one recipient strain (Krishnan and Iyer, 1988; Shintani et al., 2005; Mierzejewska et al., 2007; Brown et al., 2013; Yanagiya et al., 2018), or by determination of the kinds of bacterial isolates in which closely related plasmids are found (Fernandez-Lopez et al., 2006; Suzuki et al., 2010; Galata et al., 2018; Brooks et al., 2019). More comprehensive analyses of host ranges of plasmids were determined using microbial communities of natural environmental samples, especially for incompatibility (Inc.) P-1, P-7, P-9, and PromA plasmids (de Gelder et al., 2005; Shintani et al., 2014; Klümper et al., 2015). In these studies, culture-independent analyses of transconjugants showed that host range of plasmids could be distinguished as replication range and transfer range (Shintani et al., 2014; Klümper et al., 2015). The former is the range of hosts in which a plasmid can replicate (replication host range) and the latter is the range of hosts to which the plasmid can transfer by conjugation (transfer host range). The host range information of these studies was not only important to understand how the plasmids could promote the bacterial evolution and adaptation including the occurrence of drug resistant pathogens, but also the usage of the plasmid as a vector tool of molecular genetics. However, currently available information on the host ranges does not sufficiently cover various kinds of bacteria and plasmids.

The range of hosts in which a plasmid has replicated at some points during its evolutionary history (evolutionary host range) is of course unknown and undeterminable by experiments. Previous studies demonstrated that replicons (chromosomes and plasmids) from the same hosts tend to have similar compositions of *k*-mers or oligonucleotides such as dinucleotides (Campbell et al., 1999; Suzuki et al., 2008). The compositional similarity of replicons (chromosomes and plasmids) may be due to mutational biases homogenizing nucleotide compositions of chromosomes and plasmids [so-called ‘amelioration’ (Lawrence and Ochman, 1997)] and/or DNA exchanges between chromosomes and plasmids by recombination (Zheng et al., 2015). These facts suggest that evolutionary hosts of a plasmid could be predicted based on the similarity in oligonucleotide compositions between the plasmid and chromosomes of bacteria. Thus, oligonucleotide compositions have been used to predict evolutionary hosts of plasmids and related elements (Suzuki et al., 2010; Norberg et al., 2011; Cury et al., 2018). The evolutionary host range predicted from oligonucleotide compositions was found to be broad for broad-host-range plasmids such as IncP/P-1 and narrow for narrow-host-range plasmids such as IncF and IncI (Suzuki et al., 2010). Recently, Li et al. (2018) compared the transfer host range to the predicted evolutionary host ranges of well-known IncP/P-1 plasmids. However, to the best of our knowledge, there were no complementary studies to assess whether the evolutionary host range of the newly discovered plasmid predicted from oligonucleotide compositions (by bioinformatics) is consistent with the replication/transfer host range determined using conjugation assays (by experiments).

In the present study, we assessed host range of pSN1216-29, which was previously isolated from cow manure as a newly discovered self-transmissible plasmid used as a model plasmid (Yanagiya et al., 2018). This plasmid only has backbone genes involved in plasmid replication, maintenance, and conjugative transfer (Yanagiya et al., 2018). This is a suitable feature for the prediction of a host range because accessory genes are usually located on other mobile genetic elements, including transposons and/or integrons, which might influence the prediction (Norberg et al., 2011). The replication/transfer host range of the pSN1216-29 was experimentally determined with microbial communities extracted from soil and cow manure. The evolutionary host of the plasmid was computationally predicted based on *k*-mer compositions independently, then the results were compared to assess the extent with which the predicted evolutionary host range could coincide with the experimentally determined replication/transfer host range. In addition, compositional similarities of the plasmid-to-host (transconjugants genera) chromosomes were compared with plasmid-to-non-host (other genera) chromosomes.

## Materials and Methods

### Bacterial Strains, Plasmids, Media, and Culture Conditions

The bacterial strains used in this study are listed in Table 1. Bacterial strains were grown in Luria Broth (LB) (Sambrook and Russell, 2001) at 30°C for *Pseudomonas*, and 37°C for *Escherichia*. Antibiotics were used at 50 μg/mL for kanamycin (Km), 30 μg/mL for gentamicin (Gm), 50 μg/mL for rifampicin (Rif). The solid media were prepared by the addition of 1.5% (w/v) agar to an LB. The agar plate without any nutrients was prepared by mixing dH_2_O with 1.5% (w/v) agar, named as ‘Agar plate’.

**TABLE 1:** Bacterial strains and plasmids used in this study.

Strain or plasmid	Relevant characteristics	References
**Bacterial strains**		
*Escherichia coli*		
DH5α	F^–^, *Φ* 80d*lacZ*ΔM15, Δ(*lacZYA*-*argF*) U169, *deoR*, *recA*1, *endA*1, *hsdR*17(r_K_^–^, m_K_^+^), *phoA*, *supE*44, λ^–^, *thi*-1, *gyrA*96, *relA*1	RBC Bioscience
DH5α(mini-pBBR1MCS-3)	mini-pBBR1MCS-3-harboring DH5α, Tc^r^	This study
S17-1 λ*pir*	RK2 *tra* regulon; host for *pir*-dependent plasmids; *recA thi pro hsdR* M RP4-2-Tc:Mu-Km:Tn*7*λ*pir* Tp^r^ Sm^r^	Simon et al., 1983
*Pseudomonas putida*		
KT2440	pWW0-free *Pseudomonas putida* mt-2	Bagdasarian et al., 1981
KT2440(pBBR1MCS-5)	pBBR1MCS-5-harboring KT2440	This study
KT2440(pSN1216-29, pBBR1MCS-5)	pSN1216-29 and pBBR1MCS-5-harboring KT2440	This study
SMDBS	A *dapB*-deleted strain of SM1443, Rif^r^ of KT2440 (KT2442) with mini-Tn*5*-*lacI*^q^ cassette inserted into the chromosome	Shintani et al., 2014
SMDBS(pSN1216-29:*gfp*)	SMDBS bearing pSN1216-29:*gfp*	This study
*Pseudomonas resinovorans*		
CA10dm4RGFP	CA10dm4R (spontaneous rifampicin-resistant CA10dm4), miniTn*7*(Gm) P*_*A*__1__/O__4__/O__3_*-*gfp*-a was inserted into chromosome (Gm^r^, Cm^r^).	Yanagiya et al., 2018
CA10dm4RGFP(pSN1216-29, pBBR1MCS-2)	pSN1216-29 and pBBR1MCS-2-harboring CA10dm4RGFP	Yanagiya et al., 2018
**Plasmids**		
pBBR1MCS-2	Km^r^, *lacZ*α *mob*; compatible with IncP, IncQ, and IncW plasmids	Kovach et al., 1995
pBBR1MCS-3	Tc^r^, *lacZ*α *mob*; compatible with IncP, IncQ, and IncW plasmids	Kovach et al., 1995
mini-pBBR1MCS-3	Self-ligated 3020-bp DNA region containing *oriV*, *rep*, and Tc^r^ gene of pBBR1MCS-3	This study
pBBR1MCS-5	Gm^r^, *lacZ*α *mob*; compatible with IncP, IncQ, and IncW plasmids	Kovach et al., 1995
pJBA28	Ap^r^, Km^r^; delivery plasmid for mini-Tn*5*-Km-P*_*A*__1__/O__4__/O__3_*-RBSII-gfpmut3*-T_0_-T_1_	Andersen et al., 1998
pSN1216-29	Conjugative, broad-host-range plasmid	Yanagiya et al., 2018
pSN1216-29:*gfp*	mini-Tn*5*-Km-P*_*A*__1__/O__4__/O__3_*-RBSII-gfpmut3*-T_0_-T_1_ was inserted in the end of *tivB7* gene (25,653 nt of pSN1216-29).	This study

### Standard DNA Manipulations

Small plasmids were extracted by using NucleoSpin^®^ Plasmid EasyPure Kit (Macherey-Nagel). The total DNA of each strain (donors, recipients, and transconjugants) was extracted by using NucleoSpin^®^ Tissue Kit (Macherey-Nagel). DNAs from cow manure and the sorted cells in PBS were performed with DNeasy PowerSoil Kit (Qiagen, GmbH, Hilden, Germany). Polymerase chain reaction (PCR) was carried out on a T100^TM^ thermal cycler (Bio-Rad, Hercules, CA, United States) with the primer sets and PrimeSTAR^®^ GXL (Takara Bio) or KOD One PCR Master Mix (TOYOBO Inc.). Restriction enzymes (New England Biolabs or Takara Bio), the HiYieldTM Gel/PCR DNA fragments Extraction kit (RBC Bioscience, New Taipei City, Taiwan), NEBuilder Hifi DNA Assembly system (New England Biolabs, Ipswich, MA, United States), and competent *E. coli* DH5α cells (RBCBioscience) were employed for cloning of DNA fragments. The other procedures were performed according to standard methods (Sambrook and Russell, 2001).

### Preparation of pSN1216-29:gfp

Mini-Tn*5* with *P_*A*__1__/O__4__/O__3_*-RBSII-*gfpmut3*^∗^ and Km-resistance gene on pJBA28 (Andersen et al., 1998) was introduced into a model plasmid, pSN1216-29 (Yanagiya et al., 2018), using *E. coli* S17-1λ*pir* (Simon et al., 1983) by modified previous methods (Shintani et al., 2014, 2019). First, mini-pBBR1MCS-3, which had no *mob* and *oriT* regions of pBBR1MCS-3 (Kovach et al., 1995) was prepared as follows. PCR were performed with PrimeSTAR^®^ GXL and two sets of primers as follows: rep-oriV_F (5′-tagctgacatTATGTGGACGATGGCCGC-3′), rep-oriV_R (5′-ctggggttcgTATGATCATTTATTCTGCCTCCCAG-3′) and KmR-F (5′-aatgatcataCGAACCCCAGAGTCCCGC-3′), KmR-R (5′-cgtccacataATGTCAGCTACTGGGCTATCTGG-3′) (the nucleotides for overlapping ends during HiFi DNA assembly system were shown in lowercase). The amplification condition was: 30 cycles of 98°C for 10 s, 55°C for 15 s and 68°C for 1 min. The resultant amplicons were assembled by NEBuilder Hifi DNA Assembly system (New England Biolabs). The pSN1216-29 was transferred from *P. resinovorans* CA10dm4RGFP(pSN1216-29, pBBR1MCS-2), which was used in the exogenous plasmid capturing (Yanagiya et al., 2018), to *P. putida* KT2440(pBBR1MCS-5). Then, the resultant *P. putida* KT2440(pSN1216-29, pBBR1MCS-5) was mixed with *E. coli* S17-1λ*pir*(pJBA28). Afterward, the pSN1216-29 carrying mini-Tn*5* with *P_*A*__1__/O__4__/O__3_*-RBSII-*gfpmut3*^∗^ and Km-resistance gene in the above KT2440 was transferred to *E. coli* (mini-pBBR1MCS-3). Finally, the pSN1216-29:*gfp* was transferred from *E. coli* (pSN1216-29:*gfp*, mini-pBBR1MCS-3) to *P. putida* SMDBS. The insertion site of the *gfp* gene was 25,653 nt of pSN1216-29, in the terminal region of *tivB7* gene encoding a member of type IV secretion system (T4SS) proteins.

The presence of plasmids in the transconjugant was confirmed by PCR amplification of DNA region in each plasmid with KOD One PCR Master Mix (TOYOBO) with primers for *repA* on pSN1216-29, repA_29-F: 5′-GCCAATCAGTGACATTGTGG -3′, repA_29-R: 5′-TCACTTCCCGGTAAATCCAG-3′ (Yanagiya et al., 2018)]. The amplification was done in 30 cycles of 98°C for 10 s, 55°C for 5 s, and 68°C for 5 sec, then held at 12°C. The amplified products were subjected to agarose gel electrophoresis and confirmed their sizes.

### Collecting the Transconjugants of pSN1216-29:gfp in Microbial Communities

The donor strain, *P. putida* SMDBS(pSN1216-29:*gfp*), was precultured in LB with Km. Microbes in environmental samples including soil and cow manure were used as recipient bacteria. The soil sample was collected at Shizuoka University, Hamamatsu, Japan (34.73N 137.72E) on 5th, Jul. 2019. Extraction of microbial fraction from 40 g soil was performed as previously described (Shintani et al., 2014). The cow manure was sampled from cows that were not fed with antibiotics, in the Sumiyoshi field of the University of Miyazaki, Japan, at 11th, Oct. 2016 and 7th, Nov. 2018. The number of microbial cells in the extracted samples were counted by using microscopy after staining the cells with 4′,6-diamidino-2-phenylindole (DAPI) or SYBR Green. The mating between the donor and recipient bacteria (microbes extracted from soil samples or 1 g of cow manure) was performed as follows: One mL of overnight culture of the plasmid donor in LB-medium was harvested, washed by phosphate-buffered saline (PBS), and then suspended in PBS. Around 10^8^ colony forming units/mL (CFU/mL) of the donor suspended in 130 μL PBS was mixed with 130 μL of 10^8^∼10^9^ cells/mL bacteria extracted from the above environmental samples. The sample mixture was dropped on 0.22 μm pore-size filters on the LB agar plate for 3–6 d, or on the LB agar plate for 2 h and then the mixture was transferred to the Agar plate (without any nutrients) for 2–3 d, or on the Agar plate for 2–3 d at 30°C to collect diverse transconjugants. The mixture on the filter was re-suspended with PBS then subjected to flow cytometry and the cell sorter MoFlo XDP^®^ IntelliSort II instrument (Beckman Coulter, Denver, MA, United States) equipped with a CyClone robotic arm for plate sorting, using a 488-nm argon laser and a 70-μm nozzle orifice. The sorting of each transconjugant cell was performed under the conditions previously described (Shintani et al., 2014). In brief, the extracted bacteria from each environmental sample without donor cells were used for negative control. Based on the flow cytometry charts of negative control, we determined the gate for collecting cells with fluorescence (Supplementary Figure S1). As a culture-dependent (CD) method, each of 384 cells was sorted on LB plate by the flow cytometry and the cell sorter and incubated at 30°C for 2 d to make the cell form a colony. For a culture independent (CI) method, which could collect and analyze many cells of transconjugants, the transconjugants from cow manure, 15,000 cells of transconjugants were sorted into 100 μL PBS in a 2-mL microtube, after which their DNA were directly extracted (see section “Standard DNA Manipulations”).

### Sequencing of 16S rRNA Genes of Transconjugants

Identification of transconjugants obtained by CD method were performed by sequencing of a partial region of 16S rRNA genes by 805R primer (5′-GACTACCAGGGTATCTAATC-3′) amplified with 27F (5′-AGAGTTTGATCMTGGCTCAG-3) and 1492R (5′-TACGGYTACCTTGTTACGACTT-3) using TaKaRa ExTaq (TAKARA BIO Inc.) or KOD One (Toyobo). The conditions were: 30 cycles of 98°C for 10 s, 55°C for 30 s, and 72°C for 60 s (ExTaq), then held at 15°C or optionally, 30 cycles of 98°C for 10 s, 55°C for 5 s, and 68°C for 5 s, then held at 15°C (KOD One). The partial nucleotide sequences of the resultant PCR products were sequenced by Sanger method using 805R primer.

The 16S rRNA gene amplicon sequencing of the extracted bacterial cells from soil, cow manure samples, and that of 15,000 cells of transconjugants obtained by CI method were performed as follows. The first PCR was performed with a primer set of 515f-MIX (5′-ACACTCTTTCCCTACACGACGCTCTT CCGATCTNNNNNGTGCCAGCMGCCGCGGTAA-3′) and 806r_MIX (5′-GTGACTGGAGTTCAGACGTGTGCTCTTC CGATCTNNNNNGGACTACHVGGGTWTCTAAT-3′) using ExTaq HS (TAKARA BIO Inc.). This was setup at 94°C for 2 min, and 30 cycles of 94°C for 30 s, 50°C for 30 s, and 72°C for 30 sec, and then 72°C for 5 min. After purification of the PCR products, the second PCR was performed with a primer set of 2ndF (5′-AATGATACGGCGACCACCGAGATCTACAC-Index2-ACACT CTTTCCCTACACGACGC-3′) and 2ndR (5′-CAAGCAGAA GACGGCATACGAGAT-Index1-GTGACTGGAGTTCAGACG TGTG-3′) using ExTaq HS (TAKARA BIO). The nucleotide sequences were determined by MiSeq (2 × 300 bp, illumina San Diego, CA, United States). The read sequences matching the primer sequence were extracted using the barcode splitter of the FASTX-Toolkit^1^ and reads were trimmed with quality threshold of > 20 using sickle (Joshi and Fass, 2011^2^). All sequencing reads shorter than 40 bp were excluded from the analysis. The merge of the reads was performed using the FLASH software with a minimum overlap of 10 bp (Magoc and Salzberg, 2011). The 246-260 base reads and the above 16S rRNA gene sequences of CD method were used for identification of transconjugants by Geneious Prime 2019 software (Kearse et al., 2012) with 16S Microbial database of NCBI^3^ as the reference database. Similar nucleotide sequences were clustered into an operational taxonomic unit (OTU) based on a threshold of 97% identity.

### Software

Complete sequences of plasmids and prokaryotic chromosomes were downloaded in FASTA format using the efetch command of the EDirect software (available at https://www.ncbi.nlm.nih.gov/books/NBK179288/). The taxonomy information for each prokaryote was retrieved using TogoWS, available at http://togows.dbcls.jp/ (Katayama et al., 2010). Data analyses were implemented using R version 3.6.2^4^. Data visualization was performed using the ‘ggplot2’ package version 3.2.1 contained within the ‘tidyverse’ package version 1.2.1. All the code and scripts used in the present study are available at https://github.com/haruosuz/plasmids.

### Publicly Available Sequence Data Used

The plasmid pSN1216-29 (GenBank accession no. AP018710) and three plasmids (pKPN-704, pEC743_4, and pJHX613) were shown to be closely related with each other (Yanagiya et al., 2018). We used the three closely related plasmids and their known host chromosomes as follows: (i) plasmid pKPN-704 (NZ_CP014764) and chromosome (NZ_CP014762) from *Klebsiella pneumoniae* strain KPNIH39 (Conlan et al., 2016), (ii) plasmid pEC743_4 (NZ_CP015073) and chromosome (NZ_CP015069) from *Escherichia coli* strain Ecol_743, and (iii) plasmid pJHX613 (NZ_CP020602) and chromosome (NZ_CP020603) from *Pseudomonas aeruginosa* strain E6130952 (Xiong et al., 2017).

Refseq chromosome accessions for reference and representative prokaryotic genomes were retrieved from the National Center for Biotechnology Information (NCBI) genome list. There were 120 and 5,681 genomes in the “prok_reference_genomes.txt” and “prok_representative _genomes.txt” files, respectively, downloaded from ftp://ftp.ncbi.nlm.nih.gov/genomes/GENOME_REPORTS/at 16^th^ Jan. 2020. Of the 5,681 genomes in the “prok_representative_genomes.txt” files, 1,775 complete sequences with the Refseq chromosome sequence accessions were retained, and the remaining draft sequences with the WGS accessions were excluded from the analysis. We also included chromosomes of one representative strain randomly selected from each of the nine genera (*Buttiauxella*, *Cloacibacterium*, *Devosia*, *Gemmata*, *Labrys*, *Lelliottia*, *Raoultella*, *Rhodoplanes*, and *Sphingobacterium*) which were not included in the references and representative prokaryotic genomes mentioned above but were experimentally obtained as transconjugants (see section “RESULTS AND DISCUSSION”). There were organisms with multiple chromosomes; e.g., *Azospirillum thiophilum* strain BV-S genome consists of eight chromosomes (Fomenkov et al., 2016). In such cases, only the largest primary chromosome was retained in the analysis because the definition of secondary chromosomes and plasmids is blur (Harrison et al., 2010). Complete sequences for *Candidatus* prokaryotes (with interim taxonomic status) and a partial sequence were excluded from the analysis. The final data set included 1,887 prokaryotic chromosomes, and the sequence statistics such as length and GC content as shown in Supplementary Table S1-1.

### Measuring Distance in Oligonucleotide Composition of a Plasmid to Chromosome

The *k*-mer compositions of plasmid pSN1216-29 and its close relatives (pKPN-704, pEC743_4, and pJHX613) were compared with those of 1,887 prokaryotic chromosome DNA (885 genera) using previously described methods (Suzuki et al., 2008, 2010, 2014). Briefly, the dissimilarity in *k*-mer compositions between an entire plasmid sequence and a set of non-overlapping 5-kb chromosomal segments from one bacterial strain was measured by the Mahalanobis distance; see below and elsewhere (Suzuki et al., 2008, 2010, 2014) for details. The Mahalanobis distance (*D*^2^) of a plasmid *x* from a set of chromosomal segments with mean μ and variance-covariance matrix *S* was calculated as:

$$D^{2}={\left(x-\mu \right)}^{T}\,S^{-1}\,\left(x-\mu \right)$$

where *x* is a vector of *k*-mer abundance values for a plasmid, μ is a mean vector of *k*-mer abundance values calculated from the chromosomal segments, *S* is the variance-covariance matrix of the *k*-mer abundance values calculated from the chromosomal segments (*S*^−1^ is the inverse matrix of *S*), and the superscript *T* is the transposition operator. The Mahalanobis distance takes into account the variance-covariance structure of the oligonucleotide compositions, and is better than other distance metrics (Minkowski distance metrics such as Euclidean distance, Manhattan distance and its derivative δ-distance) in matching known plasmid-host pairs based on their compositional similarity (Suzuki et al., 2008).

The smaller the Mahalanobis distance indicates the more similar the *k*-mer compositions between a plasmid and chromosome. Because the Mahalanobis distance has no upper limit, the distance was converted to an empirical *P* value ranging from 0 (minimal similarity) to 1 (maximal similarity) as previously described (Suzuki et al., 2008). A brief explanation for the empirical *P* value is as follows: The *k*-mer compositions are calculated for an entire plasmid sequence (EP) and for each of non-overlapping 5-kb chromosomal segments (C_1_, C_2_, C_3_, …, C_n_). The Mahalanobis distance for plasmid *D*^2^(EP) is dissimilarity in *k*-mer compositions between EP and a mean (μ) of chromosomal segments (C_1_, C_2_, C_3_, …, C_n_), while the distance for chromosome *D*^2^(C) is that between each of the chromosomal segments (C_1_, C_2_, C_3_, …, C_n_) and μ. *D*^2^(C) could be *D*^2^(C_1_) (between C_1_ and μ), *D*^2^(C_2_)(C_2_ and μ), *D*^2^(C_3_)(C_3_ and μ) …or *D*^2^(C_n_)(C_n_ and μ). The empirical *P*-value is calculated from comparison between *D*^2^(EP) and the empirical distribution of *D*^2^(C). For example, a *P* value of > 0.9 indicates that the *D*^2^(EP) (between EP and μ) is smaller than > 90% of *D*^2^(C_1_) to *D*^2^(C_n_). Therefore, high *P* values of close to 1 indicate small Mahalanobis distances and similar *k*-mer compositions between a plasmid and chromosome (in detail, see Suzuki et al., 2008, 2010).

We computed a vector of *k*-mer compositions (a.k.a. oligonucleotide relative abundances) defined as *k*-mer frequencies for 2 ≤ *k* ≤ 4 normalized by mononucleotide frequencies to factor out differences in GC content using the rho statistic (Karlin and Burge, 1995; Mrázek, 2009). To take local variations in *k*-mer compositions within a chromosome into account, we used non-overlapping 5-kb chromosomal segments instead of the entire chromosomal sequence. To calculate the Mahalanobis distance, the number of chromosomal segments (as observations in the rows) must exceed the number of oligonucleotides (as variables in the columns); i.e., 16 dinucleotides or 2-mers, 64 trinucleotides or 3-mers, and 256 tetranucleotides or 4-mers. For example, the number of chromosomal segments of *Mycoplasma genitalium* G37 (NC_000908) with 580,076 bp was 116. Thus, the Mahalanobis distance for the 116 chromosomal segments from *M. genitalium* G37 cannot be calculated for the 256 tetranucleotides (4-mers). In the present study, the Mahalanobis distance for the 4, 5, and 120 of the 1,887 prokaryotic chromosomes were not available (NA) in the 2-, 3-, and 4-mer compositions, respectively (Supplementary Table S1-1).

### Statistical Analyses

We performed statistical analyses to compare the Mahalanobis distance values for plasmid-chromosome pairs between two groups of prokaryotic genera. The two comparative groups were defined based on the experimental results of conjugation assays, i.e., the genera detected as transconjugants were defined as “Transconjugants” and the other genera were as “Others.” To test the statistical significance, an asymptotic Wilcoxon-Mann-Whitney test was implemented using the ‘wilcox_test’ function in the ‘coin’ package version 1.3-1 of R. The Cliff’s Delta effect size was used to estimate the degree of overlap between two group distributions and computed using the ‘cliff.delta’ function in the ‘effsize’ package version 0.7.8 of R. A Cliff’s delta of 1.0 or -1.0 indicates the absence of overlap between the two group distributions, while 0.0 indicates that the group distributions overlap completely. A negative Cliff’s delta close to −1.0 indicates that the Mahalanobis distance values tend to be smaller in the “Transconjugants” group than in the “Others” group; i.e., that the plasmid tends to be more similar in the *k*-mer compositions to the “Transconjugants” group than to the “Others” group.

### Accession Numbers of Nucleotide Sequence Data

The partial sequences of 75 transconjugants were deposited in the DDBJ, EMBL, and GenBank databases (accession numbers LC517459 to LC517533). The amplicon sequence data of 16S rRNA genes of the sorted transconjugants in microbial communities of soil or cow manure were deposited in the DDBJ Sequence Read Archive (DRA) with accession numbers DRA009497 and DRA009498.

## Results and Discussion

### Broad Range of Transconjugants of pSN1216-29 Were Obtained by Culture-Dependent and Culture-Independent Methods

Firstly, filter mating assays were performed with pSN1216-29:*gfp* between *Escherichia coli* and *Pseudomonas putida* because the insertion site of *gfp* and Km-resistance genes were in *tivB7* gene probably encoding a member of T4SS proteins (Yanagiya et al., 2018). The insertion site was the end of the *tivB7* gene, which replaced the last three amino acid residues by two different amino acid residues, suggesting that the transferability of the plasmid might not be affected. Indeed, conjugative transfers were observed from *E. coli* to *P. putida* or vice versa.

Next, filter mating assays were performed between donor (*P. putida*) and microbes in environmental samples (cow manure or soil). The transconjugants of pSN1216-29:*gfp* were collected by flow cytometry and the cell sorter by culture-dependent (CD) and culture-independent (CI) methods, and their partial sequences of 16S rRNA genes were determined. As shown in Supplementary Table S2, 75 transconjugants obtained by CD methods (38 from cow manure and 37 from soil samples) were classified into three phyla, five classes, seven orders, eight families, and 18 genera. The amplicon sequences of the 15,000 transconjugants collected by cell sorter (CI methods) showed that they were classified in two major phyla, *Proteobacteria* and *Actinobacteria*, and six families, *Caulobacteraceae*, *Rhizobiaceae*, *Enterobacteriacea*e, *Molexellaeceae*, *Pseudomonadaceae*, and *Williamsiaceae*, although the microbial communities of soil and cow manure were different with each other (Supplementary Figure S2). The transconjugants were clustered into 169 OTUs (Supplementary Table S3). Of the 169 OTUs, eight phyla, 15 classes, 28 orders, 53 families and 66 genera were assigned, while the remaining two OTUs were not assigned to any known taxa (Supplementary Table S3). The fact that pSN1216-29 could be transferred to different phyla of bacteria showed that the host range of the plasmid was broad as previously reported (Yanagiya et al., 2018). Some transconjugants were not obtained by CD methods probably because the culture conditions for transconjugants might not be appropriate. By both methods, 76 genera were obtained as transconjugants of pSN1216-29 (Table 2).

**TABLE 2:** Lists of genera of transconjugants obtained by a culture-dependent (CD) and independent (CI) methods.

^a^Genera of transconjugants	Methods	Family	Order	Class	Phylum
*Actinomyces*	CI	*Actinomycetaceae*	*Actinomycetales*	*Actinobacteria*	*Actinobacteria*
*Brevibacterium*	CI	*Brevibacteriaceae*			
***Corynebacterium***	CI	*Corynebacteriaceae*			
*Dermacoccus*	CI	*Dermacoccaceae*			
*Gordonia*	CI	*Gordoniaceae*			
*Kocuria*	CI	*Micrococcaceae*			
*Rothia*	CI				
*Rhodococcus*	CI	*Nocardiaceae*			
*Propionibacterium*	CI	*Propionibacteriaceae*			
*Williamsia*	CI	*Williamsiaceae*			
*Chitinophaga*	CI	*Chitinophagaceae*	–	–	*Bacteroidetes*
5-7N15	CI	*Bacteroidaceae*	*Bacteroidales*	*Bacteroidia*	
*Bacteroides*	CI				
*Paludibacter*	CI	*Porphyromonadaceae*			
*Prevotella*	CI	*Prevotellaceae*			
*Aequorivita*	CI	*Flavobacteriaceae*	*Flavobacteriales*	*Flavobacteriia*	
*Chryseobacterium*	CD, CI				
*Cloacibacterium*	CI				
*Flavobacterium*	CI				
*Wautersiella*	CI				
*Fluviicola*	CI	*Cryomorphaceae*			
*Sphingobacterium*	CD, CI	*Sphingobacteriaceae*	*Sphingobacteriales*	*Sphingobacteriia*	
*Bacillus*	CD, CI	*Bacillaceae*	*Bacillales*	*Bacilli*	*Firmicutes*
*Geobacillus*	CI				
***Paenibacillus***	CI	*Paenibacillaceae*			
*Staphylococcus*	CI	*Staphylococcaceae*			
*Streptococcus*	CI	*Streptococcaceae*	*Lactobacillales*		
*Veillonella*	CI	*Veillonellaceae*			
*Leptotrichia*	CI	*Leptotrichiaceae*	*Fusobacteriales*	*Fusobacteriia*	*Fusobacteria*
***Gemmata***	CI	*Gemmataceae*	*Gemmatales*	*Planctomycetia*	*Planctomycetes*
*Mycoplana*	CI	*Caulobacteraceae*	*Caulobacterales*	*Alphaproteobacteria*	*Proteobacteria*
***Brevundimonas***	CD				
***Bradyrhizobium***	CI	*Bradyrhizobiaceae*			
***Devosia***	CI	*Hyphomicrobiaceae*			
***Hyphomicrobium***	CI				
***Rhodoplanes***	CI				
***Methylobacterium***	CI	*Methylobacteriaceae*			
***Aminobacter***	CI	*Phyllobacteriaceae*			
***Mesorhizobium***	CI				
***Agrobacterium***	CI	*Rhizobiaceae*			
*Kaistia*	CI				
***Rhizobium***	CI				
***Labrys***	CI	*Xanthobacteraceae*			
***Rhodobacter***	CI	*Rhodobacteraceae*	*Rhodobacterales*		
*Roseococcus*	CI	*Acetobacteraceae*	*Rhodospirillales*		
***Novosphingobium***	CI	*Sphingomonadaceae*	*Sphingomonadales*		
***Sphingobium***	CI				
***Sphingomonas***	CI				
***Achromobacter***	CI	*Alcaligenaceae*	*Burkholderiales*	*Betaproteobacteria*	
***Burkholderia***	CI	*Burkholderiaceae*			
***Comamonas***	CI	*Comamonadaceae*			
***Cupriavidus***	CI	*Oxalobacteraceae*			
***Janthinobacterium***	CI				
***Ralstonia***	CI				
*Shewanella*	CI	*Shewanellaceae*	*Alteromonadales*	*Gammaproteobacteria*	
*Buttiauxella*	CD	*Enterobacteriaceae*	*Enterobacteriales*		
***Cedecea***	CD				
***Citrobacter***	CI				
***Enterobacter***	CD				
***Klebsiella***	CD				
*Kluyvera*	CD				
*Lelliottia*	CD				
***Raoultella***	CD				
***Serratia***	CD, CI				
*Siccibacter*	CD				
*Yersinia*	CD				
*Acinetobacter*	CD, CI	*Moraxellaceae*	*Pseudomonadales*		
*Moraxella*	CD				
*Enhydrobacter*	CI				
*Psychrobacter*	CI				
***Pseudomonas***	CD, CI	*Pseudomonadaceae*			
***Dyella***	CI	*Xanthomonadaceae*	*Xanthomonadales*		
***Lysobacter***	CI				
***Rhodanobacter***	CI				
***Stenotrophomonas***	CI				
*Acholeplasma*	CI	*Acholeplasmataceae*	*Acholeplasmatales*	*Mollicutes*	*Tenericutes*

*^*a*^the genera found in the top 30% of the shortest Mahalanobis distances in Supplementary Table S1-1 were shown in bold.*

### Relationship of pSN1216-29 Family Plasmids and Their Known Host Chromosomes

The host in which a plasmid was found (designated as “known host”) is one of the evolutionary hosts for the plasmid. Because the plasmid pSN1216-29 was from an unknown host, we used three known hosts (*K. pneumoniae* KPNIH39, *E. coli* Ecol_743 and *P. aeruginosa* E6130952) in which the plasmids (pEC743_4, pKPN-704, and pJHX613) closely related to the pSN1216-29 were found, to model the relationships (similarities in nucleotide compositions) between these plasmids and their known hosts. Among the three known bacteria from the class *Gammaproteobacteria*, the genera *Escherichia* and *Klebsiella* (the order *Enterobacteriales*) are more closely related to each other than either one is to the genus *Pseudomonas* (the order *Pseudomonadales*) (Supplementary Table S4-1).

It has been shown that there is a positive correlation between bacterial genome length and GC content (Almpanis et al., 2018), and that plasmids generally exhibit lower GC contents relative to their hosts (Dietel et al., 2019). Among the three known hosts in which the three plasmids were found, *P. aeruginosa* E6130952 possess a larger chromosome and higher GC content (7,040,952 bp and 65.9 GC%) than *K. pneumoniae* KPNIH39 (5,351,509 bp and 57.3 GC%) and *E. coli* Ecol_743 (4,856,574 bp and 50.7 GC%) (Supplementary Table S4-1). The GC contents for the four plasmids (pSN1216-29, pEC743_4, pKPN-704, and pJHX613) ranged from 61.3 to 61.8 GC% and were thus lower than the chromosomes of *P. aeruginosa* E6130952 (65.9%) but higher than those of *K. pneumoniae* KPNIH39 (57.3%) and *E. coli* Ecol_743 (50.7%) (Supplementary Table S4-1).

The *k*-mer compositions (oligonucleotide relative abundances) were compared between the pSN1216-29 family of plasmids (pEC743_4, pKPN-704, and pJHX613) and their known host chromosomes. Based on the 2-mer composition, the four plasmids (pSN1216-29, pEC743_4, pKPN-704, and pJHX613) showed smaller Mahalanobis distance values (= higher *P*-values) with *K. pneumoniae* KPNIH39 (*P* = 0.13–0.16) than with *P. aeruginosa* E6130952 (*P* = 0.04) and *E. coli* Ecol_743 (*P* = 0.02). Based on the 3-mer and 4-mer compositions, the four related plasmids showed smaller Mahalanobis distance values (and higher *P* values) with *P. aeruginosa* E6130952 (*P* = 0.05–0.06 for 3-mer, and *P* = 0.12–0.16 for 4-mer) than with the *K. pneumoniae* KPNIH39 (*P* = 0.01 for 3-mer, *P* = 0.02–0.03 for 4-mer) and *E. coli* Ecol_743 (*P* = 0 for both 3-mer and 4-mer) (Supplementary Table S4-2). As a point of reference, the 3-mer composition of a narrow-host-range plasmid F (IncF) is highly similar to its specific host (*E. coli* within *Enterobacteriales* of *Gammaproteobacteria*, *P* = 0.91), while that of a broad-host-range plasmid RK2/RP4 (IncP/P-1) is moderately similar to diverse bacteria across three classes in *Proteobacteria*, including *Bordetella avium* of *Betaproteobacteria* (*P* = 0.75), *Mesorhizobium loti* of *Alphaproteobacteria* (*P* = 0.44), and *Pseudomonas stutzeri* of *Gammaproteobacteria* (*P* = 0.39) (Suzuki et al., 2010). Similarly, IncW plasmids, another broad-host-range plasmid group, also show relatively low *P*-values (at most, *P* = 0.12–0.31) to the bacterial strains with 3-mer composition (Suzuki et al., 2010). The low *P*-values (≤ 0.16) indicating large Mahalanobis distance and dissimilar *k*-mer compositions between the pSN1216-29 family plasmids and their known host chromosomes can be explained in at least two ways. Firstly, the plasmids recently transferred to the hosts and thus have not yet acquired the hosts’ *k*-mer compositions. Secondly, the plasmids have horizontally transferred between multiple hosts and thus their *k*-mer compositions reflect a mixture of diverse *k*-mer compositions from the multiple hosts. The second explanation suggests that the pSN1216-29 and its related plasmids have broad host ranges, consistent with the above experimental results which indicate that pSN1216-29 could be transferred to seven different phyla of bacteria (Table 2).

### Comparisons of Predicted Hosts of pSN1216-29 With Experimentally Obtained Transconjugants

We measured dissimilarities of *k*-mer compositions (*k* = 2, 3, and 4) between plasmid pSN1216-29 and 1,887 prokaryotic chromosomes (885 genera) using the Mahalanobis distance. Of the 1,887 prokaryotes, 327 bacteria (76 genera) were experimentally obtained as transconjugants by CD and/or CI methods (Supplementary Table S1-1). As shown in Table 2, the 35 of 76 genera obtained as transconjugants were listed in the top 30% of the predicted lists (453 bacteria, 252 genera, Supplementary Table S1-1). Meanwhile, top 10% of smallest Mahalanobis distances were found in 86 bacteria (56 genera) in any of the *k*-mer compositions (Supplementary Table S1-2). Among the 86 bacteria (56 genera), 26 bacteria (13 genera) including *Achromobacter*, *Aminobacter*, *Bradyrhizobium*, *Burkholderia*, *Comamonas*, *Corynebacterium*, *Cupriavidus*, *Janthinobacterium*, *Lysobacter*, *Pseudomonas*, *Ralstonia*, and *Stenotrophomonas* were obtained as transconjugants (Supplementary Table S1-2).

**FIGURE 1:**
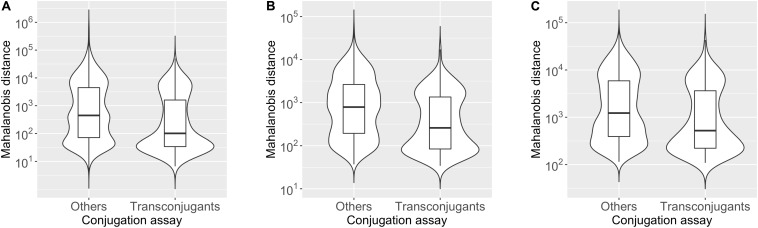
Violin box plot showing the distribution of Mahalanobis distance values of the “Transconjugants” group (*n* = 349) and “Others” group (*n* = 1,538) based on the results of conjugation assay. The dissimilarity in the *k*-mer compositions for *k* = 2 **(A)**, 3 **(B)**, and 4 **(C)** between the plasmid pSN1216-29 and prokaryotic chromosomes was measured by the Mahalanobis distance.

To assess whether the plasmid tends to have *k*-mer compositions with more similarity to hosts than non-hosts, we compared the distribution of the Mahalanobis distance values for the “Transconjugants” genera (349 chromosomes) and “Others” genera (1538 chromosomes), presented in Figure 1 as violin box plots based on 2-mer (Figure 1A), 3-mer (Figure 1B), and 4-mer (Figure 1C) compositions (see Supplementary Table S1-1 for details). The median value of the Mahalanobis distances for the plasmid-chromosome pairs was smaller in the “Transconjugants” group than in the “Others” group with Cliff’s delta effect size of −0.26, −0.30, and −0.23 based on the 2-mer, 3-mer, and 4-mer compositions, respectively. These differences were statistically significant based on an asymptotic Wilcoxon-Mann-Whitney test (2-mer *p*-value = 3.2e-14; 3-mer *p*-value < 2.2e-16; 4-mer *p*-value = 3.1e-11). This indicates that the oligonucleotide composition of the plasmid pSN1216-29 was more similar to those of the “Transconjugants” group than to those of the “Others” group, regardless of *k*-mer lengths (2, 3, and 4) used.

**FIGURE 2:**
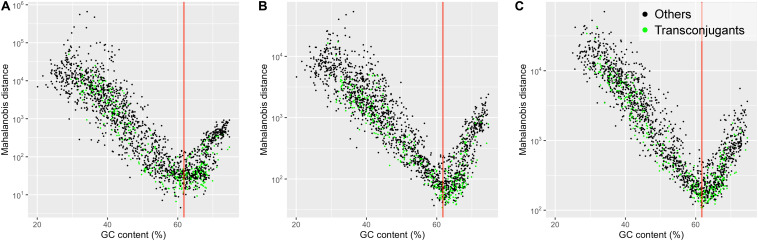
Scatter plot showing Mahalanobis distance values, plotted against GC contents for 1,887 prokaryotic chromosomes. The dissimilarity in the *k*-mer compositions for *k* = 2 **(A)**, 3 **(B)**, and 4 **(C)** between the plasmid pSN1216-29 and prokaryotic chromosomes was measured by the Mahalanobis distance. Green dots denote the chromosomes of the “Transconjugants” group and black dots denote those of the “Others” group based on the results of conjugation assay. The red vertical line indicates the value of the GC content of the plasmid pSN1216-29 (61.8%).

Figure 2 shows scatter plots of the Mahalanobis distance values plotted against GC contents for 1,887 prokaryotic chromosomes. The Mahalanobis distance values were non-linearly correlated with GC contents. The non-linear U-shaped relationship shows that the prokaryotic chromosomes with GC contents lower or higher than the GC content of the plasmid pSN1216-29 (61.8%) tended to have larger Mahalanobis distance values. Based on the 2-mer composition, the smallest Mahalanobis distance value was found in *Gammaproteobacteria*, *Halomonas subglaciescola* strain ACAM 12 (NZ_LT670847.1) with GC content of 60.8% (Figure 2A). The smallest Mahalanobis distance value was found in Betaproteobacteria, *Bordetella avium* 197N (NC_010645.1) with GC content of 61.6% based on 3-mer compositions (Figure 2B), while the smallest value was found in *Alphaproteobacteria*, *Bradyrhizobium erythrophlei* strain GAS242 (NZ_LT670818.1) with GC content of 61.9% based on 4-mer compositions (Figure 2C). Thus, prokaryotic chromosomes with GC contents similar to the GC content of the plasmid pSN1216-29 (61.8%) tended to have small Mahalanobis distances indicating similar *k*-mer compositions to the plasmid pSN1216-29, regardless of *k*-mer lengths (2, 3, and 4) used. Note that the *k*-mer compositions (oligonucleotide relative abundances) were normalized to factor out differences in GC content (Karlin and Burge, 1995; Mrázek, 2009). A possible explanation for the plasmid-chromosome compositional similarity by the process called “amelioration” (Lawrence and Ochman, 1997, 1998) is that all replicons (plasmids and chromosomes) in the same host have been subjected to host-specific mutational biases and that the replicons have acquired the host’s compositional features such as GC content and *k*-mer compositions.

We must acknowledge that there might be false positives and false negatives for the experimental results of determining transconjugants based on conjugation assay and/or evolutionary host prediction based on *k*-mer compositions. For the conjugation assay, an example of false positives is that some bacteria might have autofluorescence (Yang et al., 2012), although few cells showed autofluorescence in the negative control of our samples (Supplementary Figure S1). The example of false negatives is that some transconjugants could not be detected because GFP is not expressed in a functional manner in the cell (Overkamp et al., 2013). Based on *k*-mer compositions, plasmid pSN1216-29 may show low similarity with some chromosomes from the evolutionary hosts (false negatives), while the plasmid may show high similarity with non-hosts’ chromosomes (false positives). Because some distantly related prokaryotes have similar *k*-mer compositions (Mrázek, 2009), the plasmid may have similar *k*-mer compositions with non-hosts by chance. It should be noted that the Mahalanobis distances in *k*-mer compositions between the plasmid and chromosomes varied even in the same genus (Supplementary Figure S3). This was because the *k*-mer compositions varied among bacterial taxa even within the same genus (van Passel et al., 2006).

It will be necessary to determine the whole genome sequences of transconjugants themselves for more accurate evaluation of compositional similarity between the plasmid and host chromosomes. This allows more accurate comparisons between the evolutionary host range and replication/transfer host range of the plasmid. In addition, various factors have been found that determine or affect the host range of a plasmid within itself and/or in its host chromosome, including DNA polymerase, helicase, gyrase, and nucleoid associated proteins involved in the capability of replication, maintenance, and/or conjugation of a plasmid (Shintani et al., 2015; Shintani and Suzuki, 2019; Yano et al., 2019). Therefore, it is necessary to consider the absence or presence of these factors on the plasmid or chromosomes for the prediction of plasmid host range.

## Conclusion

The goal of the present study was to test whether the evolutionary host range predicted computationally is consistent with the replication/transfer host range determined experimentally. The various kinds of transconjugants of different phyla experimentally obtained in this study clearly showed that the newly discovered conjugative plasmid pSN1216-29 has a broad host range. The oligonucleotide compositions between the plasmid and its host (transconjugants genera) chromosomes were more similar than those between the plasmid and non-host (other genera) chromosomes. These findings indicate that the evolutionary host range of the plasmid is partly consistent with its replication/transfer host range. For more accurate comparisons, nucleotide sequences of transconjugants themselves remain to be determined and compared to those of the plasmid. The accurate prediction of plasmid host range will shed light on the understanding of how plasmids behave in microbial communities, and also in designing appropriate plasmid vectors for different bacteria.

## Data Availability Statement

The datasets generated for this study can be found in the DDBJ, EMBL, and GenBank databases: LC517459 to LC517533, DDBJ Sequence Read Archive: DRA009497 and DRA009498.

## Author Contributions

MS and HS conceived, designed, and supervised the study. MT, HS, KY, MY, and MS performed the experiments and data analysis. HS, KI, MY, MO, KK, and MS wrote, reviewed, and edited the manuscript. All authors contributed to manuscript revision, read and approved the submitted version.

## Conflict of Interest

The authors declare that the research was conducted in the absence of any commercial or financial relationships that could be construed as a potential conflict of interest.

## Footnotes

1 http://hannonlab.cshl.edu/fastx_toolkit/

2 https://github.com/najoshi/sickle

3 https://ftp.ncbi.nlm.nih.gov/blast/db/

4 https://www.r-project.org/
