# Supplementary material for: Determination of Plasmid pSN1216-29 Host Range and the Similarity in Oligonucleotide Composition Between Plasmid and Host Chromosomes
Source: Front Microbiol. 2020 Jun 9;11:1187. doi: 10.3389/fmicb.2020.01187 (PMC7296055; doi:10.3389/fmicb.2020.01187)
Supplement: Supplementary file 1 [file Presentation_1.PPTX]

## Slide 1
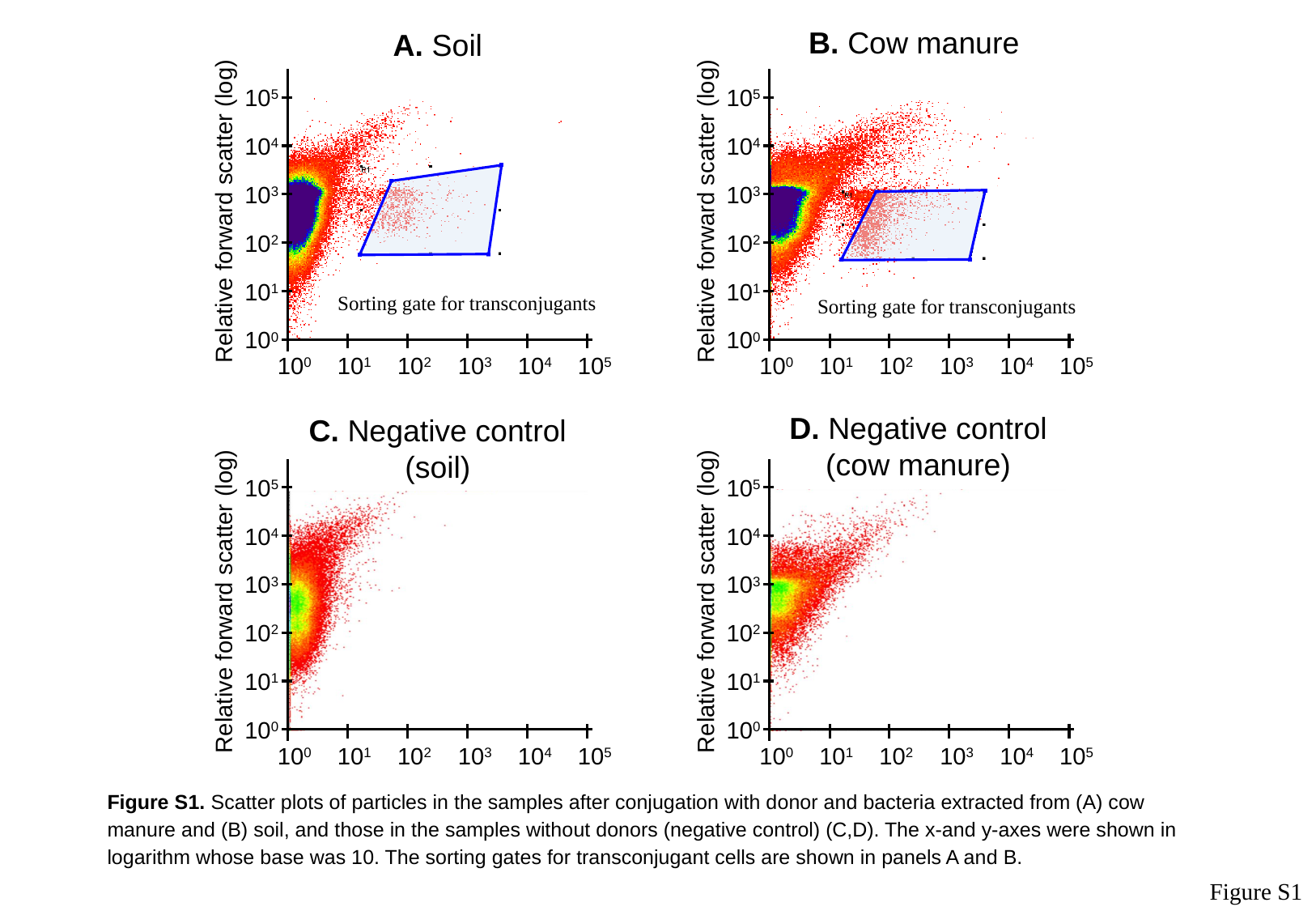

B. Cow manure
A. Soil
105
104
103
Relative forward scatter (log)
102
101
100
100
101
102
103
104
105
Sorting gate for transconjugants
105
104
103
Relative forward scatter (log)
102
101
100
100
101
102
103
104
105
Sorting gate for transconjugants
D. Negative control
(cow manure)
C. Negative control
(soil)
105
104
103
Relative forward scatter (log)
102
101
100
100
101
102
103
104
105
105
104
103
Relative forward scatter (log)
102
101
100
100
101
102
103
104
105
Figure S1. Scatter plots of particles in the samples after conjugation with donor and bacteria extracted from (A) cow manure and (B) soil, and those in the samples without donors (negative control) (C,D). The x-and y-axes were shown in logarithm whose base was 10. The sorting gates for transconjugant cells are shown in panels A and B.
Figure S1

## Slide 2
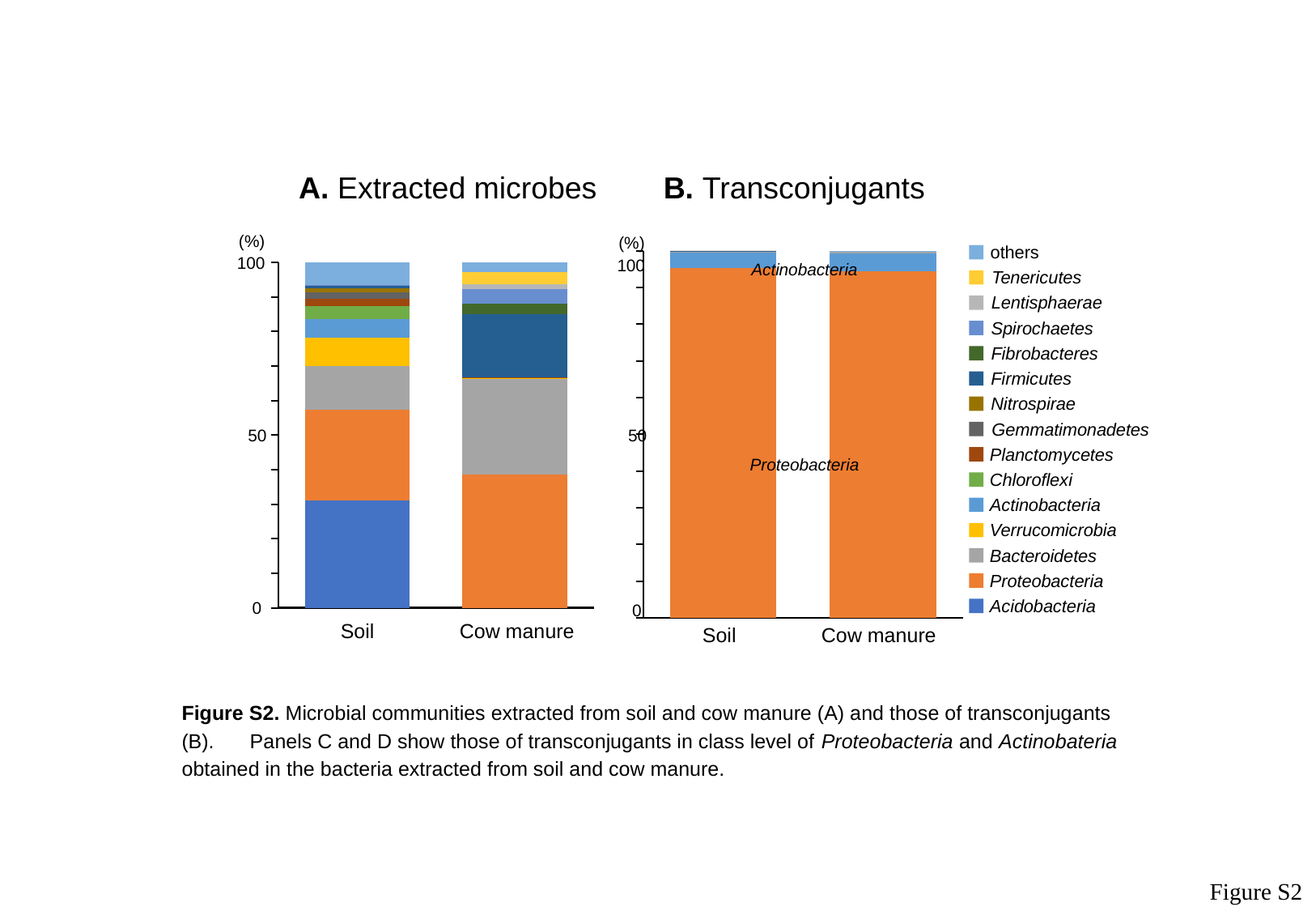

A. Extracted microbes
B. Transconjugants
(%)
(%)
others
Tenericutes
Lentisphaerae
Spirochaetes
Fibrobacteres
Firmicutes
Nitrospirae
Gemmatimonadetes
Planctomycetes
Chloroflexi
Actinobacteria
Verrucomicrobia
Bacteroidetes
Proteobacteria
Acidobacteria
### Chart
| Category | Proteobacteria | Actinobacteria | Bacteroidetes | Firmicutes | Planctomycetes | Tenericutes | Others |
|---|---|---|---|---|---|---|---|
| 合計 / Soil_sum | 124648.0 | 5610.0 | 371.0 | 173.0 | 13.0 | 0.0 | 2.0 |
| 合計 / Cow_sum | 112241.0 | 5778.0 | 599.0 | 178.0 | 0.0 | 72.0 | 63.0 |
### Chart
| Category | Acidobacteria | Proteobacteria | Bacteroidetes | Verrucomicrobia | Actinobacteria | Chloroflexi | Planctomycetes | Gemmatimonadetes | Nitrospirae | Firmicutes | Fibrobacteres | Spirochaetes | Lentisphaerae | Tenericutes | Others |
|---|---|---|---|---|---|---|---|---|---|---|---|---|---|---|---|
| soil% | 31.02662834210098 | 26.29209826997126 | 12.747437496610445 | 8.040023862465427 | 5.474808829112208 | 3.8234177558435922 | 2.085254081023917 | 1.9496718911003852 | 1.0656760127989586 | 0.7592602635717772 | 0.0894842453495309 | 0.0325397255816476 | 0.0 | 0.0 | 6.613699224469876 |
| cow_manure% | 0.0 | 38.636244974677595 | 27.692789641309457 | 0.19057066778050435 | 0.16446509685166816 | 0.0 | 0.010442228371534485 | 0.0 | 0.0 | 18.255625750535167 | 2.994308985537514 | 4.276092518143372 | 1.5062914425938494 | 3.461598705163682 | 2.81156998903566 |100
100
Actinobacteria
50
50
Proteobacteria
0
0
Soil
Cow manure
Soil
Cow manure
Figure S2. Microbial communities extracted from soil and cow manure (A) and those of transconjugants (B). 　Panels C and D show those of transconjugants in class level of Proteobacteria and Actinobateria obtained in the bacteria extracted from soil and cow manure.
Figure S2

## Slide 3
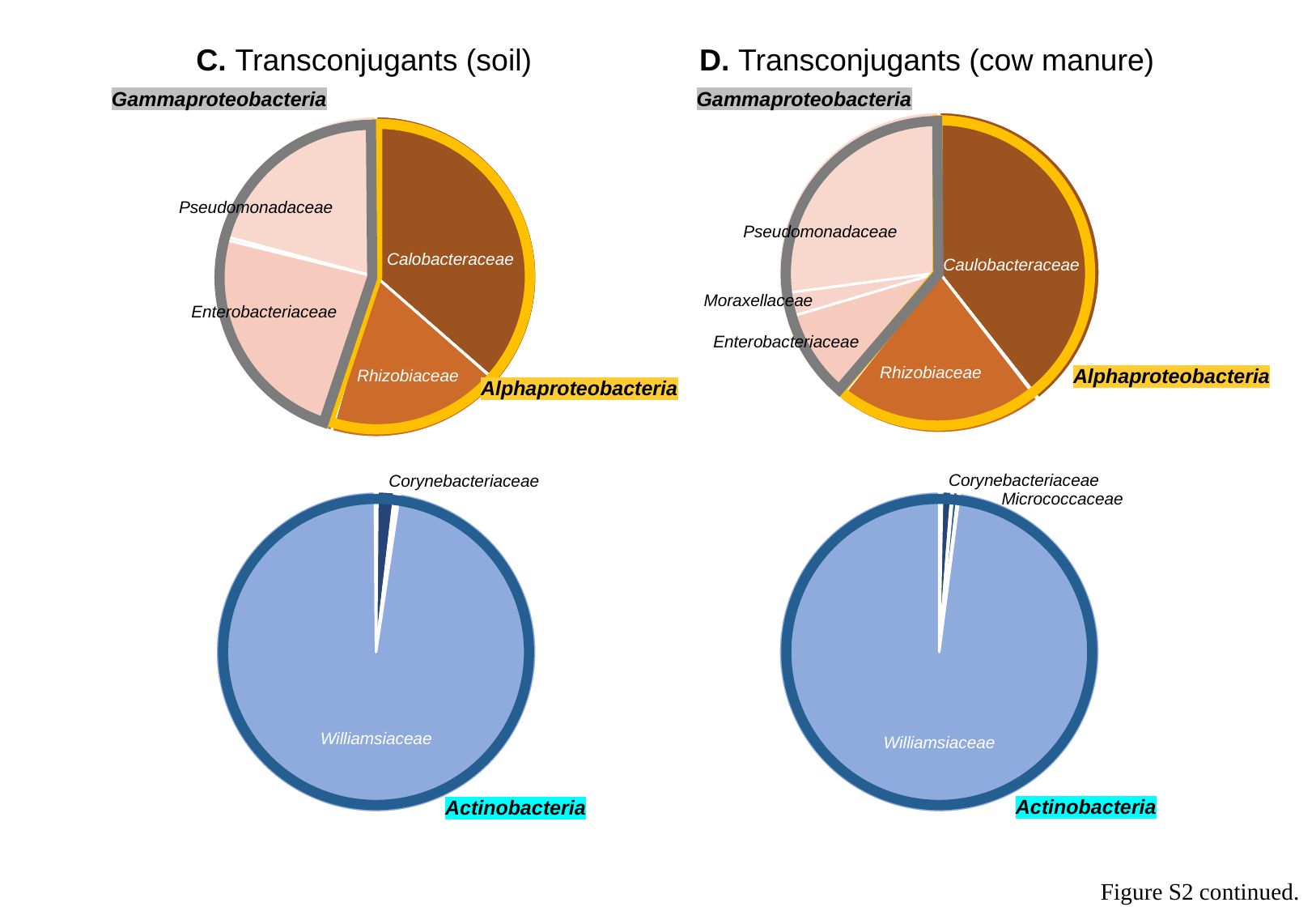

C. Transconjugants (soil)
D. Transconjugants (cow manure)
Gammaproteobacteria
Gammaproteobacteria
### Chart
| Category | |
|---|---|
| Alphaproteobacteria | None |
| Caulobacterales | None |
| Caulobacteraceae | 0.37147589778947454 |
| Ellin329 | None |
| (空白) | 0.0 |
| Rhizobiales | None |
| Bartonellaceae | 0.00013453178733887716 |
| Bradyrhizobiaceae | 0.0 |
| Brucellaceae | 0.00022702239113435522 |
| Hyphomicrobiaceae | 0.0007315165936551446 |
| Methylobacteriaceae | 0.00015134826075623682 |
| Phyllobacteriaceae | 9.249060379547806e-05 |
| Rhizobiaceae | 0.1990565958412861 |
| Xanthobacteraceae | 0.0 |
| (空白) | 0.005768050382154359 |
| Rhodobacterales | None |
| Rhodobacteraceae | 0.0 |
| Rhodospirillales | None |
| Acetobacteraceae | 0.0 |
| Rickettsiales | None |
| mitochondria | 7.56741303781184e-05 |
| Sphingomonadales | None |
| Sphingomonadaceae | 0.00032792123163851306 |
| Betaproteobacteria | None |
| Burkholderiales | None |
| Alcaligenaceae | 0.0014041755303495305 |
| Burkholderiaceae | 0.0 |
| Comamonadaceae | 0.0011519284290891357 |
| Oxalobacteraceae | 0.0005969848063162675 |
| Neisseriales | None |
| Neisseriaceae | 0.00035314594176455254 |
| Gammaproteobacteria | None |
| Aeromonadales | None |
| Aeromonadaceae | 5.0449420252078936e-05 |
| Alteromonadales | None |
| Shewanellaceae | 0.00046245301897739023 |
| Enterobacteriales | None |
| Enterobacteriaceae | 0.08236708679822755 |
| Pseudomonadales | None |
| Moraxellaceae | 0.023408530996964622 |
| Pseudomonadaceae | 0.2554001900261496 |
| Xanthomonadales | None |
| Xanthomonadaceae | 0.0005129024392294692 |
### Chart
| Category | |
|---|---|
| Alphaproteobacteria | None |
| Caulobacterales | None |
| Caulobacteraceae | 0.34678979031777213 |
| Ellin329 | None |
| (空白) | 6.11541313437856e-05 |
| Rhizobiales | None |
| Bartonellaceae | 0.0 |
| Bradyrhizobiaceae | 8.408693059770519e-05 |
| Brucellaceae | 0.0 |
| Hyphomicrobiaceae | 7.644266417973199e-05 |
| Methylobacteriaceae | 0.0 |
| Phyllobacteriaceae | 0.00015288532835946398 |
| Rhizobiaceae | 0.17180488774394762 |
| Xanthobacteraceae | 0.00011466399626959798 |
| (空白) | 0.005763776879151792 |
| Rhodobacterales | None |
| Rhodobacteraceae | 5.3509864925812394e-05 |
| Rhodospirillales | None |
| Acetobacteraceae | 3.8221332089865995e-05 |
| Rickettsiales | None |
| mitochondria | 0.0 |
| Sphingomonadales | None |
| Sphingomonadaceae | 0.0010701972985162477 |
| Betaproteobacteria | None |
| Burkholderiales | None |
| Alcaligenaceae | 0.0003669247880627136 |
| Burkholderiaceae | 0.00031341492313690115 |
| Comamonadaceae | 0.0007414938425434003 |
| Oxalobacteraceae | 3.05770656718928e-05 |
| Neisseriales | None |
| Neisseriaceae | 0.0005274543828401508 |
| Gammaproteobacteria | None |
| Aeromonadales | None |
| Aeromonadaceae | 0.0001758181276133836 |
| Alteromonadales | None |
| Shewanellaceae | 0.0 |
| Enterobacteriales | None |
| Enterobacteriaceae | 0.2229832514122782 |
| Pseudomonadales | None |
| Moraxellaceae | 0.0033634772239082076 |
| Pseudomonadaceae | 0.1980935199553575 |
| Xanthomonadales | None |
| Xanthomonadaceae | 0.00023697225895716916 |
Pseudomonadaceae
Pseudomonadaceae
Calobacteraceae
Caulobacteraceae
Moraxellaceae
Enterobacteriaceae
Enterobacteriaceae
Rhizobiaceae
Alphaproteobacteria
Rhizobiaceae
Alphaproteobacteria
Corynebacteriaceae
Corynebacteriaceae
Micrococcaceae
### Chart
| Category | |
|---|---|
| Acidimicrobiia | None |
| C111 | 2.0 |
| (空白) | 6.0 |
| Actinobacteria | None |
| Actinomycetaceae | 0.0 |
| Brevibacteriaceae | 0.0 |
| Corynebacteriaceae | 96.0 |
| Dermacoccaceae | 5.0 |
| Gordoniaceae | 0.0 |
| Microbacteriaceae | 15.0 |
| Micrococcaceae | 9.0 |
| Nocardiaceae | 0.0 |
| Propionibacteriaceae | 0.0 |
| Williamsiaceae | 5471.0 |
| (空白) | 6.0 |
### Chart
| Category | |
|---|---|
| Acidimicrobiia | None |
| C111 | 0.0 |
| (空白) | 0.0 |
| Actinobacteria | None |
| Actinomycetaceae | 9.0 |
| Brevibacteriaceae | 8.0 |
| Corynebacteriaceae | 55.0 |
| Dermacoccaceae | 0.0 |
| Gordoniaceae | 8.0 |
| Microbacteriaceae | 0.0 |
| Micrococcaceae | 29.0 |
| Nocardiaceae | 4.0 |
| Propionibacteriaceae | 5.0 |
| Williamsiaceae | 5660.0 |
| (空白) | 0.0 |
Williamsiaceae
Williamsiaceae
Actinobacteria
Actinobacteria
Figure S2 continued.

## Slide 4
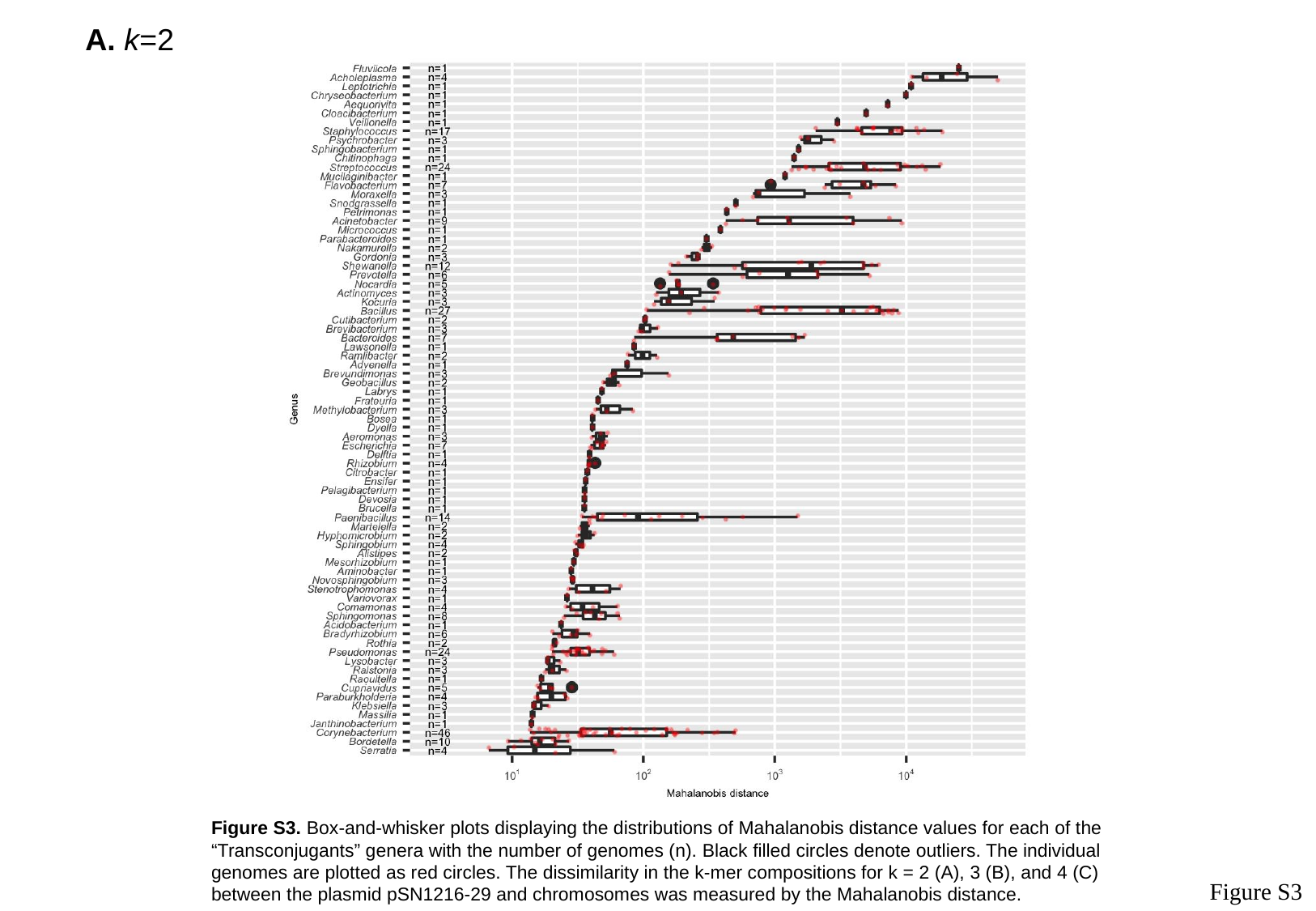

A. k=2
Figure S3. Box-and-whisker plots displaying the distributions of Mahalanobis distance values for each of the “Transconjugants” genera with the number of genomes (n). Black filled circles denote outliers. The individual genomes are plotted as red circles. The dissimilarity in the k-mer compositions for k = 2 (A), 3 (B), and 4 (C) between the plasmid pSN1216-29 and chromosomes was measured by the Mahalanobis distance.
Figure S3

## Slide 5
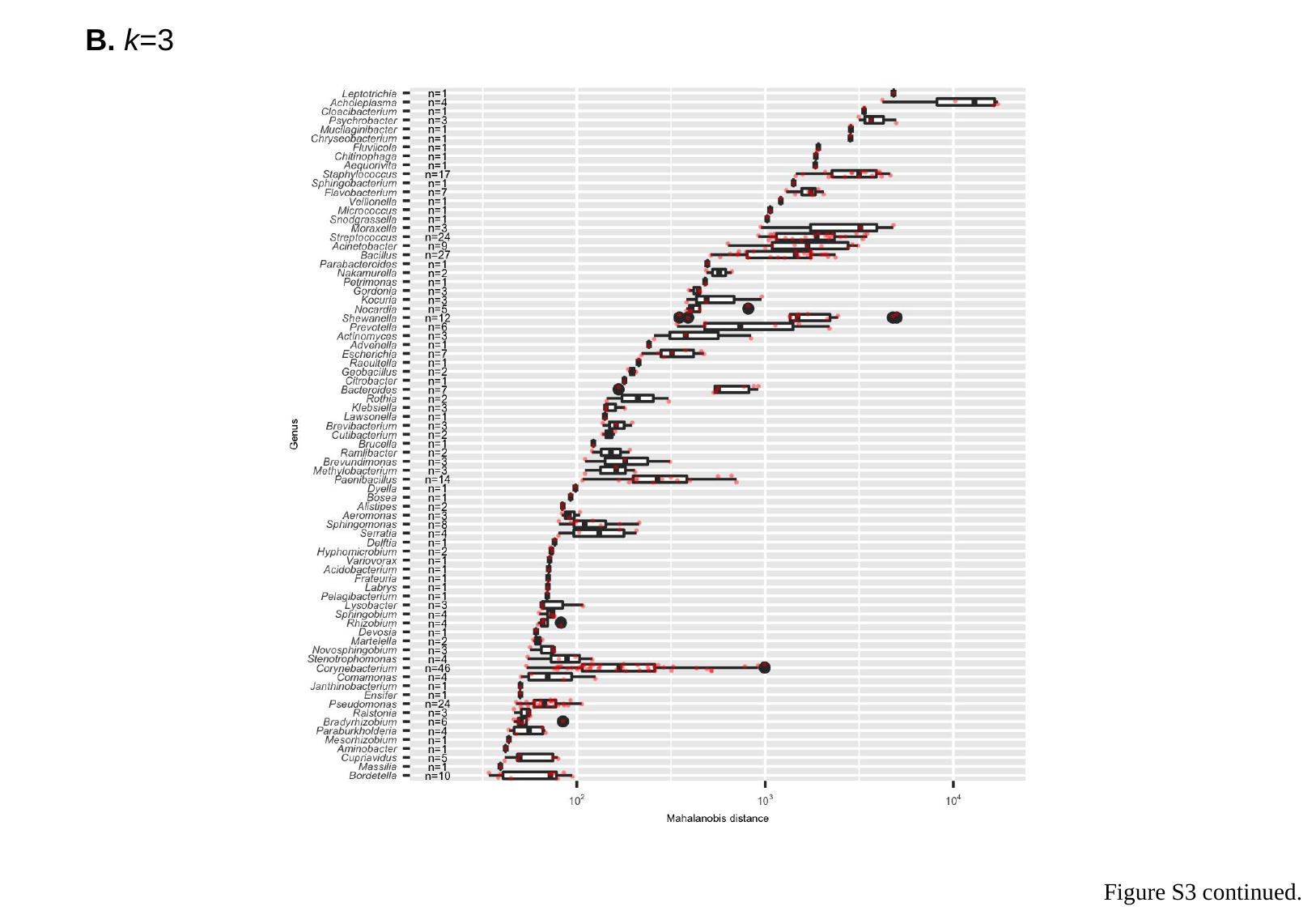

B. k=3
Figure S3 continued.

## Slide 6
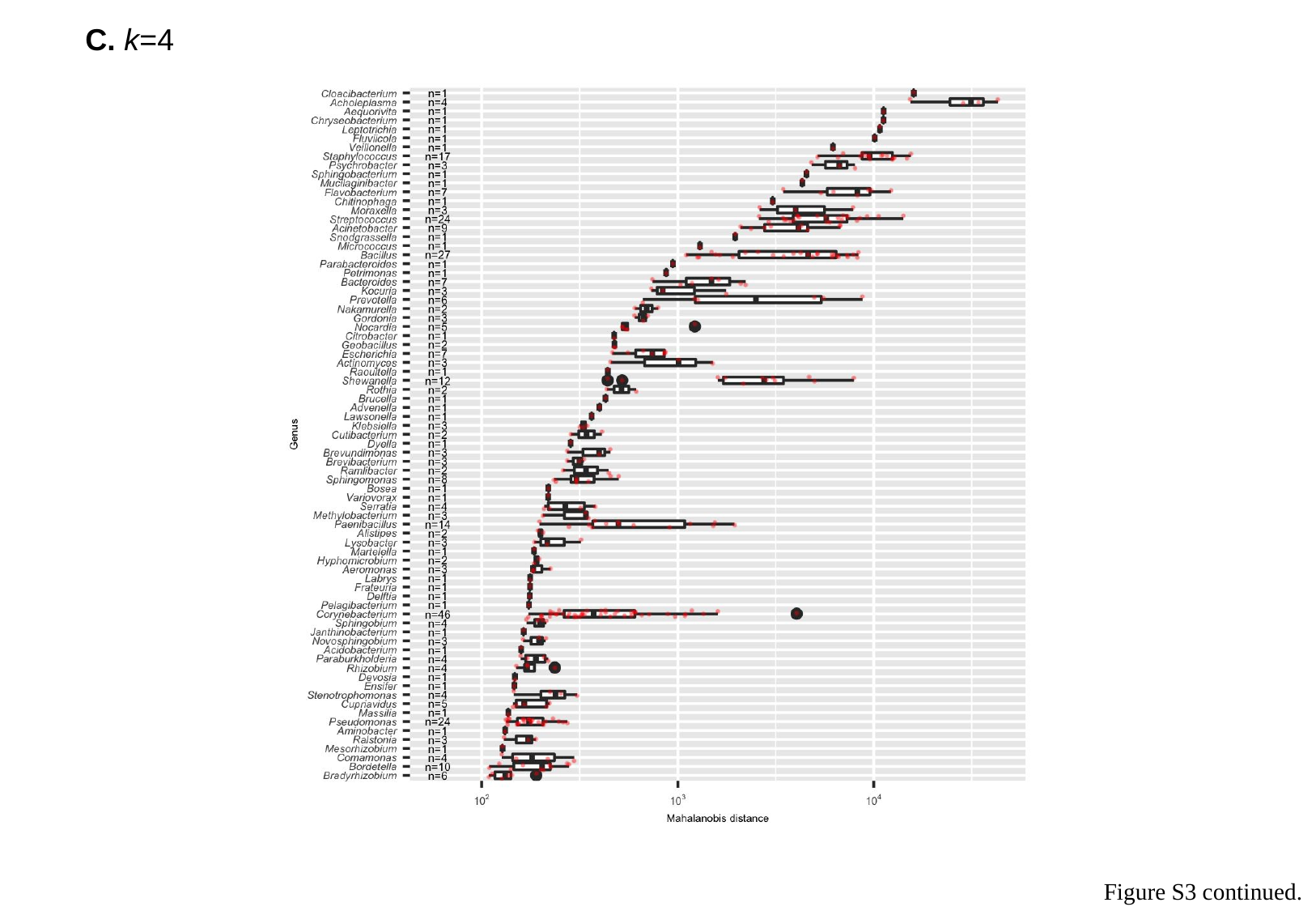

C. k=4
Figure S3 continued.
